# Hedgehog acyltransferase catalyzes a random sequential reaction and utilizes multiple fatty acyl-CoA substrates

**DOI:** 10.1016/j.jbc.2022.102422

**Published:** 2022-08-24

**Authors:** Adina R. Schonbrun, Marilyn D. Resh

**Affiliations:** 1 Cell Biology Program, Memorial Sloan Kettering Cancer Center, New York, New York, USA; 2 Gerstner Sloan Kettering Graduate School, New York, New York, USA; 3 Biochemistry, Cell Biology and Molecular Biology Graduate Program, Weill-Cornell Graduate School of Medical Sciences, New York, New York, USA

**Keywords:** hedgehog acyltransferase, sonic hedgehog, palmitoylation, MBOAT, fatty acyl-CoA, enzyme kinetics, hedgehog signaling, DGAT1, Diacylglycerol acyltransferase 1, ER, endoplasmic reticulum, Hhat, hedgehog acyltransferase, MBOAT, membrane-bound *O*-acyltransferase, Porcn, Porcupine, Ptch, Patched, Shh, Sonic hedgehog, Smo, Smoothened

## Abstract

Sonic hedgehog (Shh) signaling is a key component of embryonic development and is a driving force in several cancers. Hedgehog acyltransferase (Hhat), a member of the membrane-bound *O*-acyltransferase family of enzymes, catalyzes the attachment of palmitate to the N-terminal cysteine of Shh, a posttranslation modification critical for Shh signaling. The activity of Hhat has been assayed in cells and *in vitro*, and cryo-EM structures of Hhat have been reported, yet several unanswered questions remain regarding the enzyme’s reaction mechanism, substrate specificity, and the impact of the latter on Shh signaling. Here, we present an *in vitro* acylation assay with purified Hhat that directly monitors attachment of a fluorescently tagged fatty acyl chain to Shh. Our kinetic analyses revealed that the reaction catalyzed by Hhat proceeds through a random sequential mechanism. We also determined that Hhat can utilize multiple fatty acyl-CoA substrates for fatty acid transfer to Shh, with comparable affinities and turnover rates for myristoyl-CoA, palmitoyl-CoA, palmitoleoyl-CoA, and oleoyl-CoA. Furthermore, we investigated the functional consequence of differential fatty acylation of Shh in a luciferase-based Shh reporter system. We found that the potency of the signaling response in cells was higher for Shh acylated with saturated fatty acids compared to monounsaturated fatty acids. These findings demonstrate that Hhat can attach fatty acids other than palmitate to Shh and suggest that heterogeneous fatty acylation has the potential to impact Shh signaling in the developing embryo and/or cancer cells.

Hedgehog (Hh) proteins, most prominently exemplified by Sonic hedgehog (Shh), are secreted morphogens that mediate cell signaling crucial to development (1, 2). In the Shh signaling pathway, Shh binds to Patched (Ptch) on the cell surface, alleviating Ptch-mediated inhibition of Smoothened (Smo) and culminating in activation of Gli transcription factors to promote transcription of Shh target genes (3). Prior to secretion, Shh proteins undergo multiple processing steps in the endoplasmic reticulum (ER) (4). The N-terminal signal sequence is cleaved, and autocleavage of the 45 kDa precursor protein produces an N-terminal 19 kDa fragment as well as a 26 kDa C-terminal portion that is degraded. Two lipid modifications then occur to generate the mature, 19 kDa Shh protein. Cholesterol is linked to the C terminus, a reaction coupled to autoproteolysis, and Hh acyltransferase (Hhat) catalyzes attachment of palmitate to the N-terminal cysteine (5, 6, 7). Hhat is a multipass, ER-localized membrane protein and is a member of the membrane-bound *O*-acyltransferase (MBOAT) family of enzymes. Studies in flies and mice have demonstrated that palmitoylation is critical for cellular signaling (8, 9). Activation of the Shh pathway has been implicated in multiple cancers (10, 11), and therefore, understanding how Hhat activates this pathway is of great clinical interest.

To date, several assays have been reported to measure the catalytic activity of Hhat. However, these assays employ radioactive substrates (6, 12) or require specialized equipment (13) or multiple steps for product readout (14). The most recently reported *in vitro* Hhat assay that avoids these issues employed Hhat in solubilized membranes and utilized fluorescence anisotropy of an acylated Shh peptide for assay readout (15, 16). The advantage of the assay we present in this study is that it is the first direct, fluorescent assay that tracks the fatty acyl group attached to Shh to measure catalytic activity of purified Hhat.

Two recent cryo-EM studies provide major insights into the structure of the active site and the transmembrane topology of Hhat and propose a molecular mechanism for Hhat-mediated Shh palmitoylation (12, 17). However, the kinetic mechanism of Hhat catalysis—the sequence of substrate binding and product release—remains unknown, not only for Hhat but also for other MBOAT enzymes including Diacylglycerol acyltransferase 1 (DGAT1), ghrelin *O*-acyltransferase, and Porcupine (Porcn). Unlike for DHHC acyltransferases (18), no study has reported whether Hhat catalysis involves an acyl-enzyme intermediate or instead proceeds *via* formation of a ternary complex.

Another unresolved aspect of Hhat biology concerns fatty acyl-CoA substrate specificity. Palmitoyl-CoA is thought to be the primary acyl-CoA substrate for Hhat, yet fatty acids other than palmitate have been detected on Shh purified from mammalian cells, in some cases at even higher proportions than palmitate (19, 20). The ability of Hhat to directly transfer fatty acids other than palmitate to Shh has not been reported. The fatty acid selectivity of Hhat is important because it has the potential to play a critical role in Shh signaling. When different fatty acids and hydrophobic groups are artificially attached to Shh by nonenzymatic *in vitro* acylation, the strength of Shh signaling output increases with increasing chain length and hydrophobicity (21, 22). However, investigation of the effect of unsaturation on signaling through fatty acylated Shh has been limited and indirect; only one study showed that Shh isolated from chick limb buds exhibits reduced signaling activity when the explants are incubated with palmitoleate compared to palmitate (19).

To address the aforementioned issues, we used our *in vitro* Shh palmitoylation assay to determine the kinetic mechanism of Hhat catalysis and investigate Hhat’s substrate specificity. We also further explored the consequences of differential fatty acylation on Shh signaling directly in cells. We report that Hhat catalyzes a random sequential reaction, that Hhat can attach both saturated and unsaturated fatty acids of multiple chain lengths to Shh, and that Shh signaling in cells is more potent when Shh is modified with a saturated fatty acid compared to unsaturated fatty acids.

## Results

### Development of a direct, fluorescent assay to measure Hhat activity

**Figure 1:**
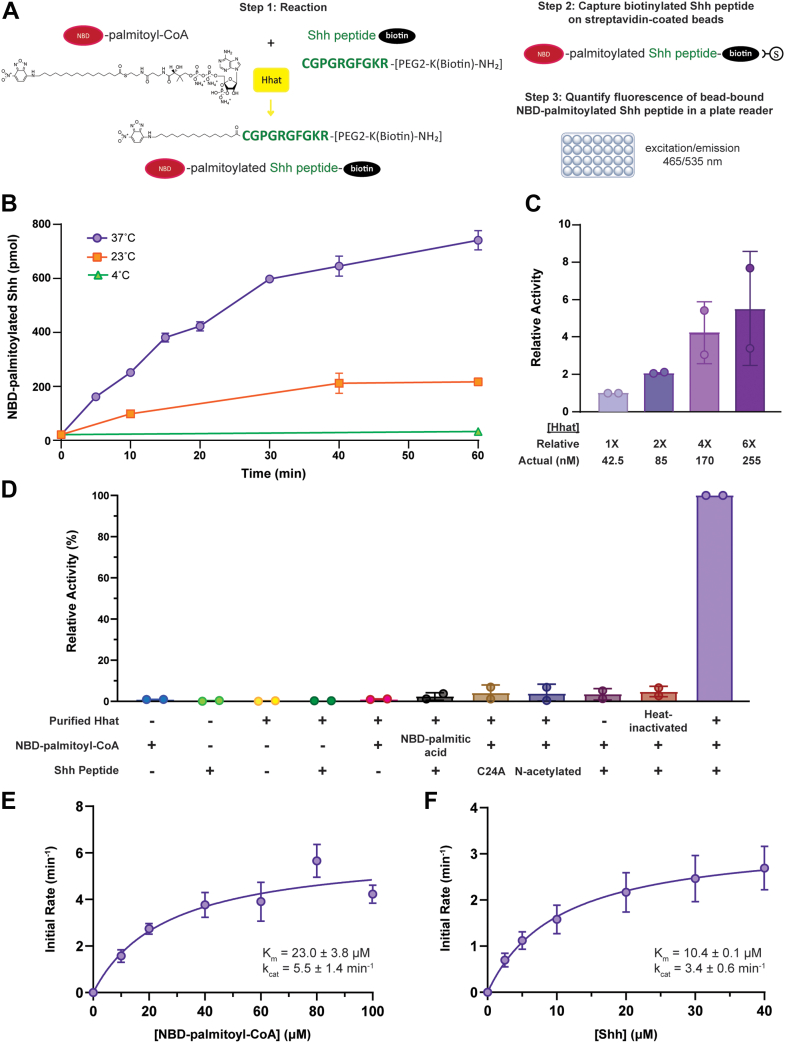
***In vitro* assay for Hhat-mediated Shh fatty acylation.***A*, assay schematic. *B*, 25 μM NBD-palmitoyl-CoA and 25 μM WT Shh peptide were incubated with purified Hhat as indicated. NBD-palmitoylated Shh was quantified, with subtraction of background fluorescence from the average of duplicate 60-min reactions containing elution buffer instead of Hhat. This experiment was performed at least twice (n ≥ 2) in duplicate. A representative graph is shown. Data points represent the average of duplicate reactions ± SD. *C*, 25 μM NBD-palmitoyl-CoA and 25 μM WT Shh peptide were incubated with the indicated concentrations of purified Hhat at 37 °C for 10 min. NBD-palmitoylated Shh was quantified, with subtraction of background fluorescence from the average of duplicate reactions containing elution buffer instead of 255 nM Hhat. Data were normalized to that of the reactions with 42.5 nM Hhat. This experiment was performed at least twice (n ≥ 2) in duplicate. Data points represent the average of two experiments ± SD. *D*, reactions containing elution buffer or purified Hhat (untreated or heat-inactivated at 95 °C for 10 min), 10 μM NBD-palmitoyl-CoA or NBD-palmitic acid, and/or 10 μM WT, C24A, or N-acetylated Shh peptide were incubated for 40 min at 37 °C. NBD-palmitoylated Shh was quantified, and data normalized to that of the standard reaction. Each reaction condition was assayed at least twice (n ≥ 2) in duplicate. Data points represent the average of two assays ± SD. *E*, increasing concentrations of NBD-palmitoyl-CoA were incubated with 25 μM WT Shh peptide and purified Hhat or elution buffer for 10 min at 37 °C. The initial rate of Hhat-mediated NBD-palmitoylation of Shh was quantified; Michaelis-Menten fit of the data is shown with apparent kinetic parameters as insets. Data points represent the average of at least two experiments (n ≥ 2) performed in duplicate, ± SD. *F*, As in *E*, except WT Shh peptide was titrated in the presence of 25 μM NBD-palmitoyl-CoA. Hhat, hedgehog acyltransferase; Shh, sonic hedgehog.

**Figure 2:**
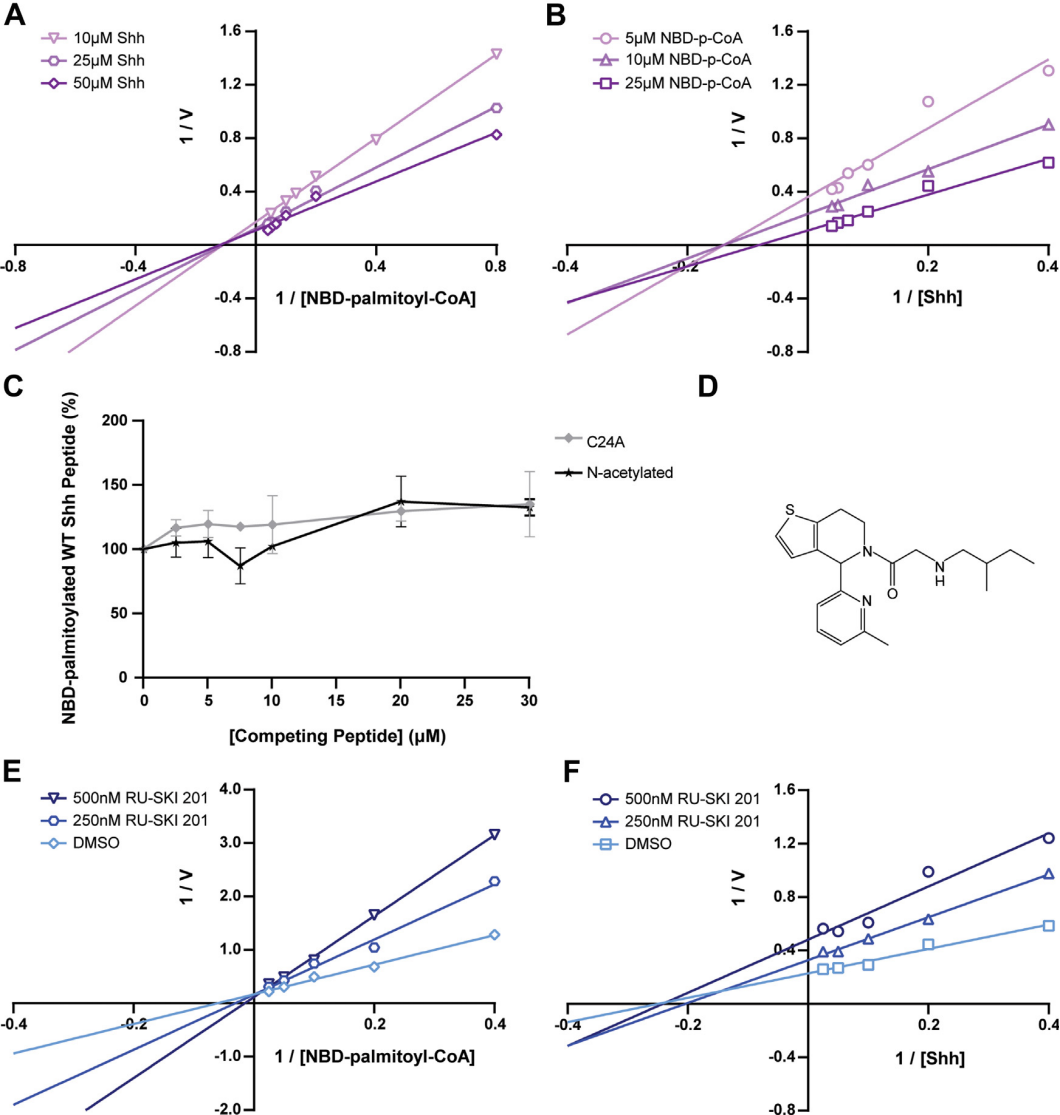
**Kinetic mechanism of Hhat.***A*, increasing concentrations of NBD-palmitoyl-CoA were incubated with purified Hhat or elution buffer and the indicated concentrations of WT Shh peptide for 10 min at 37 °C. The initial rate of Hhat-mediated NBD-palmitoylation of Shh was quantified; Lineweaver–Burk plots of the data are shown. Each line contains the average data points from at least two titrations (n ≥ 2) performed in duplicate. *B*, as in *A*, except WT Shh peptide was titrated in the presence of the indicated concentrations of NBD-palmitoyl-CoA. *C*, purified Hhat, 10 μM NBD-palmitoyl-CoA, and 10 μM WT Shh peptide were incubated with increasing concentrations of N-acetylated and C24A Shh peptides for 10 min at 37 °C. NBD-palmitoylated Shh was quantified, and data were normalized to the amount of NBD-palmitoylated Shh when no competing peptide was present. This experiment was performed twice (n = 2) in duplicate. Data points represent the average of two experiments ± SD. *D*, chemical structure of RU-SKI 201. *E*, increasing concentrations of NBD-palmitoyl-CoA were incubated with 10 μM WT Shh peptide, purified Hhat or elution buffer, and 0 (DMSO), 250, and 500 nM RU-SKI 201 for 10 min at 37 °C. The initial rate of Hhat-mediated NBD-palmitoylation of Shh was quantified; Lineweaver–Burk plots of the data are shown. Each titration was performed at least twice (n ≥ 2) in duplicate. A representative graph is shown. *F*, as in *E*, except WT Shh peptide was titrated in the presence of 0 (DMSO), 250, and 500 nM RU-SKI 201. Each line contains the average data points from two titrations (n = 2) performed in duplicate. Hhat, hedgehog acyltransferase; Shh, sonic hedgehog.

We sought to develop a direct, fluorescent assay to measure the acyltransferase activity of Hhat (Fig. 1*A*). The assay utilizes a reaction mix containing purified Hhat (6, 23), a biotinylated Shh peptide containing 10 N-terminal amino acids sufficient for fatty acylation (24), and NBD-palmitoyl-CoA, a fluorescent analog of palmitoyl-CoA that serves as a substrate for other fatty acyltransferases (25, 26, 27, 28, 29). Following incubation, the biotinylated Shh peptide was captured on streptavidin-coated agarose beads, and fluorescence intensity of the NBD-palmitoylated Shh product was quantified. Palmitoylation of Shh occurred in a time-, temperature-, and enzyme-dependent manner (Fig. 1, *B* and *C*), and a stoichiometry of 0.99 ± 0.05 mol palmitate/mol Shh peptide was achieved. Substitution of the N-terminal cysteine on the Shh peptide with alanine or blockage with N-acetylation reduced the fluorescent signal to background, as did use of a free fatty acid (NBD-palmitate) instead of NBD-palmitoyl-CoA (Fig. 1*D*). Hhat activity was also strongly reduced when the enzyme was heat-inactivated (Fig. 1*D*) or when a small molecule Hhat inhibitor, RU-SKI 201 (Fig. 2*D*) (30), was included in the reaction (Fig. 2, *E* and *F*). Hhat-mediated Shh palmitoylation obeyed Michaelis–Menten kinetics (Fig. 1, *E* and *F*), with apparent kinetic values similar to those reported previously (6, 12, 14).

### Kinetic mechanism of Hhat catalysis

Bisubstrate enzyme reactions typically proceed through one of two mechanisms (31). In a nonsequential (ping-pong) reaction, one substrate binds, an enzyme-intermediate is formed, and the first product is simultaneously released. The second substrate then binds, and the second product is formed and released. Alternatively, in a sequential reaction, both substrates need to be bound to the enzyme in a ternary complex in order for catalysis to occur, resulting in simultaneous release of both products. In both cases, the enzyme’s affinity for its substrates and/or maximal turnover rate can change with increasing concentrations of one fixed substrate, until saturating concentrations of the fixed substrate are reached. Sequential *versus* nonsequential kinetic mechanisms can be distinguished by titrating one substrate in the presence of increasing, nonsaturating concentrations of the second substrate. In Lineweaver–Burk plots of 1/V *versus* 1/[S], intersecting lines indicate a sequential reaction, whereas parallel lines indicate a nonsequential reaction (Table 1). As depicted in Figure 2, *A* and *B*, intersecting lines were observed when Shh was titrated in the presence of increasing concentrations of NBD-palmitoyl-CoA, and vice versa. These findings rule out a ping-pong mechanism and suggest that the kinetic mechanism of Hhat-mediated acylation of Shh is *via* formation of a ternary complex in a sequential reaction.

**Table 1:** Differentiating features in Lineweaver–Burk plots for kinetic mechanism

Titration experiment	Nonsequential	Random sequential	Ordered sequential: Palmitoyl-CoA binds before Shh	Ordered sequential: Shh binds before palmitoyl-CoA
Differentiating a nonsequential *versus* random or ordered sequential reaction				
Titrating palmitoyl-CoA in the presence of various [fixed Shh]	Parallel lines	Intersecting lines to the left of the Y-axis	Intersecting lines to the left of the Y-axis, above, or below the X-axis	Intersecting lines on the Y-axis
Titrating Shh in the presence of various [fixed palm-CoA]	Parallel lines	Intersecting lines to the left of the Y-axis	Intersecting lines on the Y-axis	Intersecting lines to the left of the Y-axis, above, or below the X-axis
Differentiating a random versus ordered sequential reaction using an Hhat inhibitor competitive with palmitoyl-CoA				
Titrating palmitoyl-CoA in the presence of various [fixed inhibitor]		Intersecting lines on the Y-axis		
Titrating Shh in the presence of various [fixed inhibitor]		Intersecting lines to the left of the Y-axis	Intersecting lines on the Y-axis	Parallel lines

Adapted from Table VI-1, Ref (31).

Sequential reactions can occur in a random fashion, in which either substrate can bind before the other or in an ordered fashion, in which one substrate is required to bind first in order for the second substrate to bind. Random *versus* ordered sequential reactions can be differentiated with Lineweaver–Burk plots from the substrate titrations performed above (Fig. 2, *A*, *B* and Table 1). In a random reaction, the Lineweaver–Burk plots for both titrations will yield intersecting lines to the left of the Y-axis. In an ordered reaction, the Lineweaver–Burk plot for titration of the second substrate in the presence of increasing concentrations of the obligate first substrate will yield intersecting lines along the Y-axis. Lineweaver–Burk plots titrating NBD-palmitoyl-CoA or Shh in the presence of increasing concentrations of the other yielded intersecting lines to the left of the Y-axis (Fig. 2, *A* and *B*), indicative of a random sequential reaction (Table 1).

To confirm this finding, we implemented an additional approach. Titration of one substrate with various fixed concentrations of a dead-end inhibitor competitive with the other substrate will yield patterns of lines in Lineweaver–Burk plots that indicate whether the reaction is random or ordered (31). To identify a competitive, dead-end Hhat inhibitor, we first investigated potential inhibitory properties of the N-acetylated and C24A Shh peptides. However, these peptides were neither substrates (Fig. 1*D*) nor inhibitors (Fig. 2*C*) of Hhat. We then tested RU-SKI 201 (Fig. 2*D*) for potential competition with palmitoyl-CoA. Titration of palmitoyl-CoA in the presence of various fixed concentrations of RU-SKI 201 produced intersecting lines on the Y-axis of a Lineweaver–Burk plot (Fig. 2*E*), indicative of a competitive inhibition profile. Of note, a derivative of RU-SKI 201, IMP-1575, was found to inhibit Hhat by competing with palmitoyl-CoA binding (17, 32). When Shh was titrated in the presence of various fixed concentrations of RU-SKI 201, intersecting lines to the left of the Y-axis were observed (Fig. 2*F*), ruling out a mechanism whereby either substrate is required to bind first and confirming a random sequential kinetic mechanism (Table 1).

### Fatty acyl-CoA substrate specificity of Hhat

**Figure 3:**
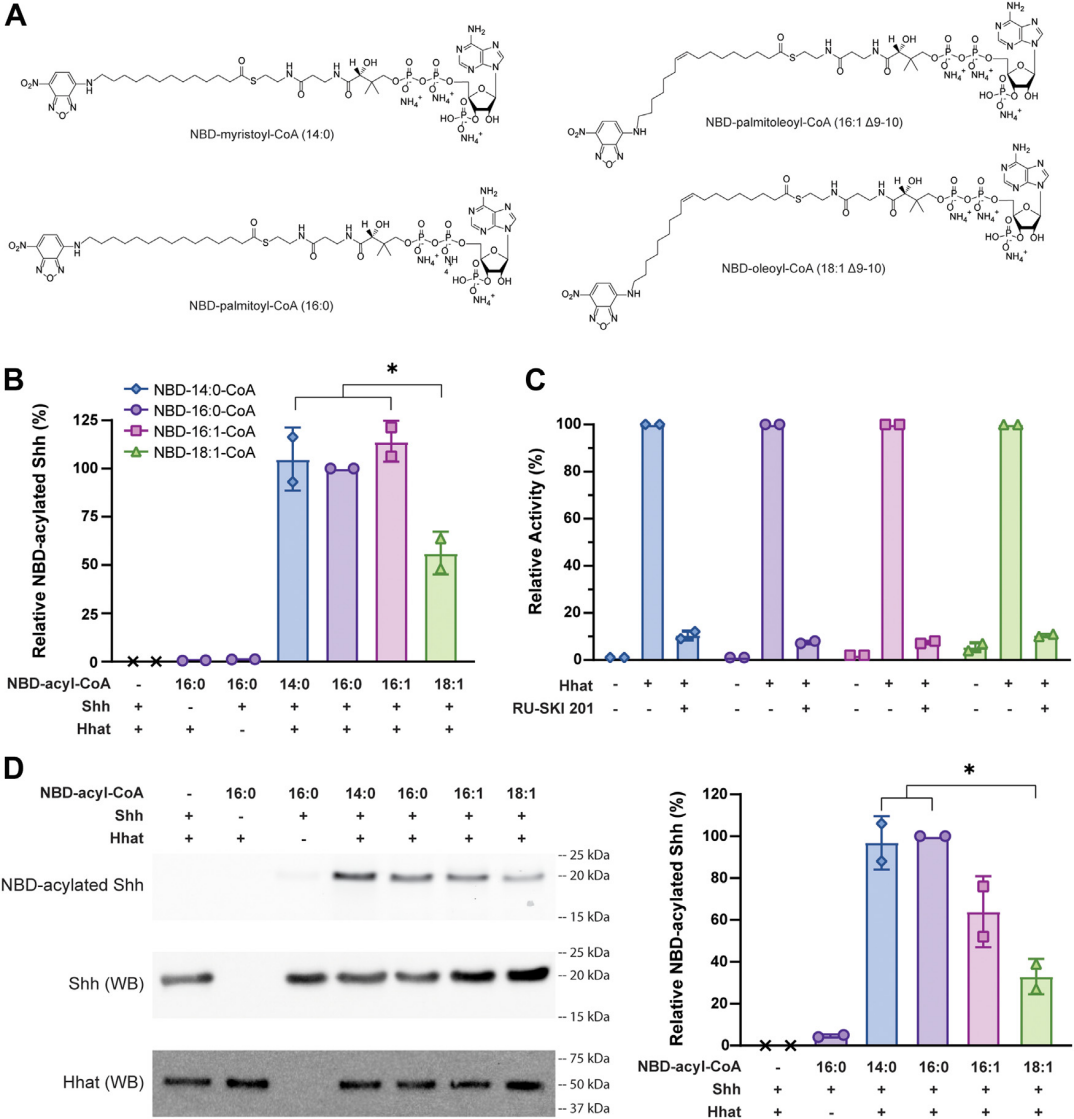
**Fatty acyl-CoA substrate specificity of Hhat**. *A*, chemical structures of NBD-labeled fatty acyl-CoAs. *B*, purified Hhat or elution buffer, 10 μM NBD-fatty acyl-CoA, and/or 10 μM WT Shh peptide were incubated for 1 h at 37 °C. NBD-fatty acylated Shh was quantified, and data were normalized to that of the NBD-palmitoyl-CoA reaction. Data points represent the average of two experiments (n = 2) performed in duplicate ± SD. Significant differences (∗*p* ≤ 0.05) were determined *via* ordinary one-way ANOVA with Tukey's multiple comparisons test. *C*, purified Hhat or elution buffer, 10 μM NBD-fatty acyl-CoA, and 10 μM WT Shh peptide were incubated with or without 10 μM RU-SKI 201 for 40 min at 37 °C. NBD-fatty acylated Shh was quantified, and data were normalized to that of the untreated reaction for each NBD-fatty acyl-CoA. Data points represent the average of two experiments (n = 2) performed in duplicate ± SD. *D*, purified Hhat or elution buffer, 10 μM NBD-acyl-CoA, and/or 10 μM recombinant, 19 kDa Shh protein were incubated for 1 h at 37 °C, and samples were subjected to PAGE. NBD fluorescence was detected by fluorescent imaging, and Shh and Hhat proteins were detected by Western blot. This experiment was performed four times. NBD-fluorescence intensity values were normalized for levels of Shh in each sample. Data were normalized to that of the NBD-palmitoyl-CoA reaction. Data points represent the average of two experiments ± SD. Hhat, hedgehog acyltransferase; Shh, sonic hedgehog.

We next investigated the ability of Hhat to catalyze transfer of fatty acids other than palmitate to Shh. Four NBD-labeled fatty acyl-CoAs were chosen: NBD-myristoyl-CoA (14:0), NBD-palmitoyl-CoA (16:0), NBD-palmitoleoyl-CoA (16:1 Δ9-10), and NBD-oleoyl-CoA (18:1 Δ9-10) (Fig. 3*A*), the primary substrates of the fatty acyltransferases N-myristoyltransferase, DHHC acyltransferases, Porcn, and DGAT1/2 and acyl-CoA cholesterol acyltransferase 1/2, respectively. We first examined fatty acid incorporation into Shh as the reaction approached completion. Similar overall amounts of myristate, palmitate, and palmitoleate were transferred to the Shh peptide, but the amount of oleate transferred was significantly lower compared to myristate and palmitoleate (Fig. 3*B*). Incorporation of all four NBD-labeled fatty acids was strongly and equally inhibited by RU-SKI 201 (Fig. 3*C*). Hhat-mediated transfer of fatty acids to the recombinant, 19 kDa Shh protein was then examined by analyzing the reaction products *via* SDS-PAGE. The pattern of NBD-fatty acid incorporated into Shh protein was similar to that observed for the Shh peptide, with lowest incorporation of oleate into Shh and no other significant differences observed (Fig. 3*D*).

**Figure 4:**
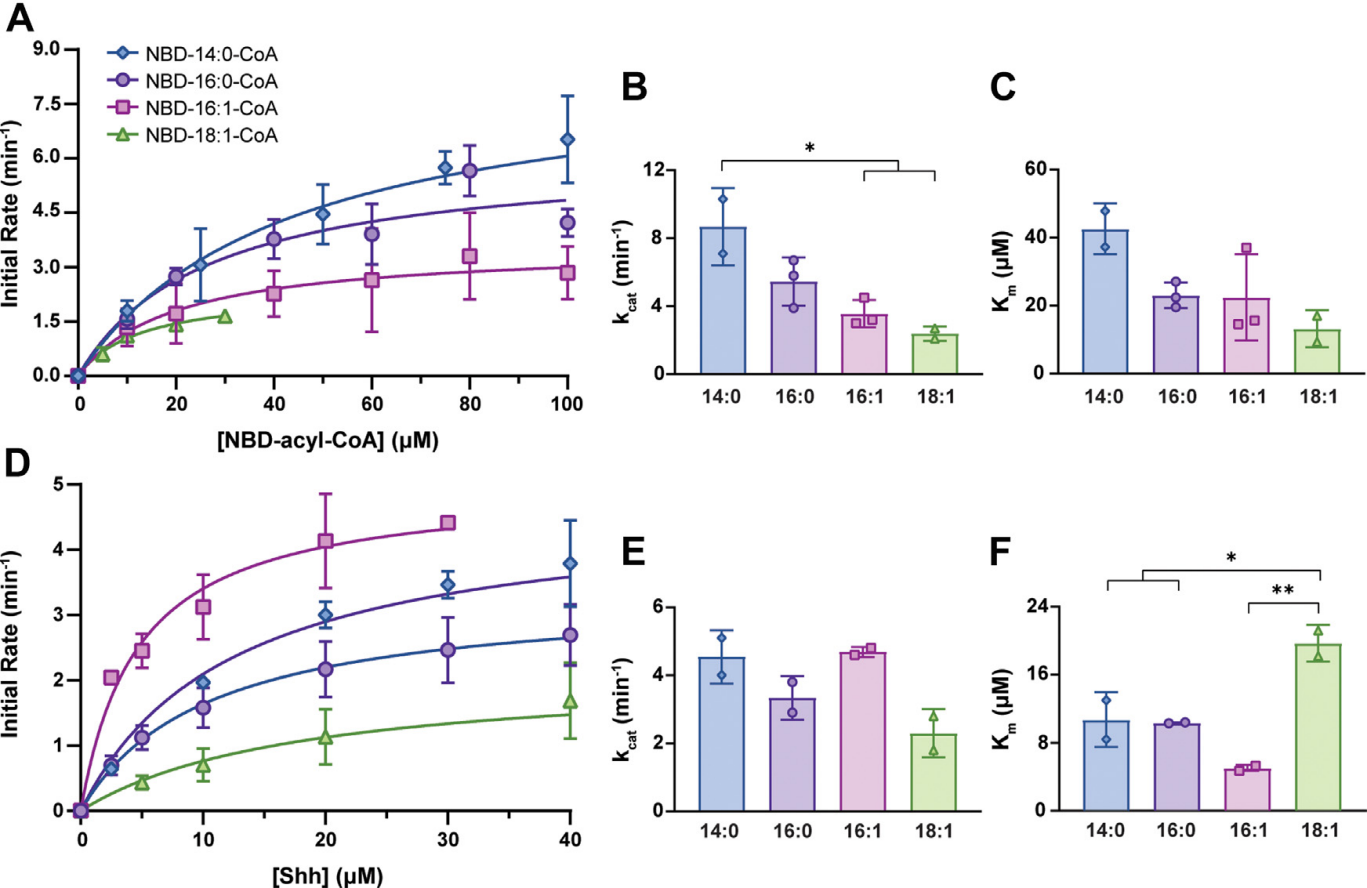
**Kinetic analysis of Hhat fatty acyl-CoA substrate specificity.***A*, NBD-fatty acyl-CoAs were incubated with 25 μM WT Shh peptide and purified Hhat or elution buffer for 10 min at 37 °C. The initial rate of Hhat-mediated NBD-fatty acylation of Shh was quantified; Michaelis–Menten fit of the data is shown. Data obtained when NBD-oleoyl-CoA was titrated above 30 μM did not continue to fit the Michaelis–Menten model, possibly due to alteration in substrate accessibility conferred by a monomer to micelle transition (the critical micelle concentration is 32–33 μM in phosphate buffer (53, 54)). Data points from each curve represent the average of at least two titrations (n ≥ 2) performed in duplicate, ± SD. *B and C*, apparent kinetic values (*B*, K_m_; *C*, k_cat_) for Hhat with the four NBD-acyl-CoAs were derived from the Michaelis–Menten fit of the data. Values represent the averages (±SD) of at least two titrations (n ≥ 2) performed in duplicate (per NBD-acyl-CoA). Significant differences are noted with *asterisks* (∗*p* ≤ 0.05, ∗∗*p* ≤ 0.01) and were determined *via* ordinary one-way ANOVA multiple comparisons. *D*, as in *A*, except WT Shh peptide was titrated in the presence of 25 μM NBD-acyl-CoA. *E and F*, as in *B* and *C*. Hhat, hedgehog acyltransferase; Shh, sonic hedgehog.

To further explore potential differences in fatty acyl-CoA substrate specificity, we employed initial rate studies for kinetic analysis of Hhat. Each of the four NBD-acyl-CoAs was titrated individually in the presence of the same fixed concentration of Shh peptide (Fig. 4*A*) and vice versa (Fig. 4*D*), and apparent K_m_ and k_cat_ values were determined (Fig. 4, *B*, *C*, *E*, *F* and Table 2). Titration of the NBD-acyl-CoAs yielded similar kinetic values for Hhat with all four fatty acyl-CoAs, with the exception of a higher turnover rate (k_cat_) with myristoyl-CoA compared to palmitoleoyl-CoA and oleoyl-CoA (Fig. 4, *B*, *C* and Table 2). Titration of Shh in the presence of the same fixed concentration of each NBD-acyl-CoA revealed similar turnover rates among all four NBD-acyl-CoAs (Fig. 4*E* and Table 2). Hhat exhibited a lower affinity (higher K_m_) for Shh in the presence of oleoyl-CoA compared to the other three acyl-CoAs but exhibited no significant differences in affinity for Shh among the other acyl-CoAs (Fig. 4*F* and Table 2). These data demonstrate that Hhat can transfer multiple types of fatty acids, both saturated and monounsaturated, to Shh.

**Table 2:** Hhat apparent kinetic parameters

Titrating NBD-acyl-CoA in the presence of 25 μM Shh		
NBD-acyl-CoA	K_m_ (μM NBD-acyl-CoA)	k_cat_ (min^−1^)
Myristoyl (14:0)	42.5 ± 7.5	8.7 ± 2.3
Palmitoyl (16:0)	23.0 ± 3.8	5.5 ± 1.4
Palmitoleoyl (16:1)	22.4 ± 12.7	3.6 ± 0.8
Oleoyl (18:1)	13.2 ± 5.4	2.4 ± 0.4

Titrating Shh in the presence of 25 μM NBD-acyl-CoA		
NBD-acyl-CoA	K_m_ (μM Shh)	k_cat_ (min^−1^)
Myristoyl (14:0)	10.7 ± 3.3	4.6 ± 0.8
Palmitoyl (16:0)	10.4 ± 0.1	3.4 ± 0.6
Palmitoleoyl (16:1)	5.0 ± 0.4	4.7 ± 0.1
Oleoyl (18:1)	19.7 ± 2.1	2.3 ± 0.7

Reported apparent kinetic values represent the averages (±SD) of at least two titrations (n ≥ 2) performed in duplicate (per NBD-acyl-CoA).

### Consequences of differential fatty acylation of Shh on cellular signaling

**Figure 5:**
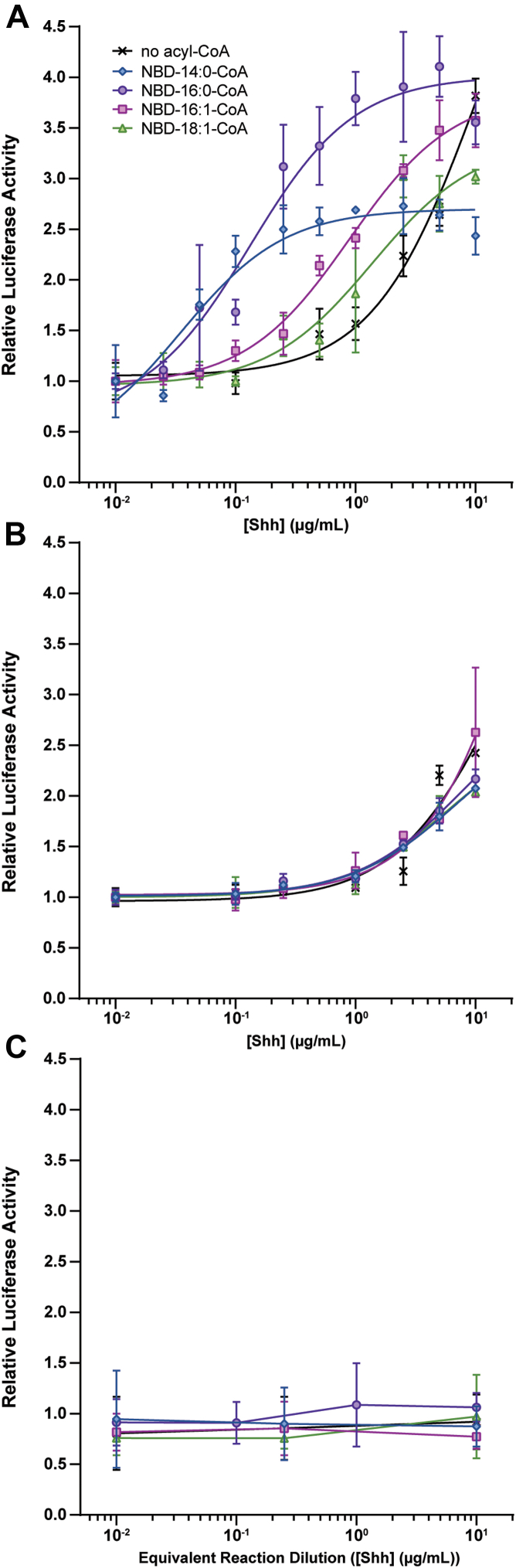
**Differential fatty acylation of Shh regulates Shh signaling activity.***A*, Shh Light II cells were treated with increasing amounts of NBD-fatty acylated Shh protein (19 kDa) from Hhat, NBD-acyl-CoA, and Shh-containing reactions, and relative signaling output was quantified. Data for each reaction were normalized to the average Firefly/*Renilla* luciferase value at 0.01 μg/ml Shh. Data points represent the average of three titrations (n = 3) performed in duplicate ± SD. *B*, As in *A*, except cells were incubated with equivalent dilution preparations from reactions lacking Hhat. Data points represent the average of two titrations (n = 2) performed in duplicate ± SD. *C*, as in *A*, except cells were incubated with equivalent dilution preparations from reactions lacking Shh protein. Data were normalized to the average Firefly/*Renilla* luciferase value of untreated wells. Data points represent the average of two titrations (n = 2) performed in duplicate, ± SD. Hhat, hedgehog acyltransferase; Shh, sonic hedgehog.

Palmitoylation of Shh plays a key role in propagation of Shh signaling through Ptch (20, 33). Recent structural studies of Ptch1 have revealed that the palmitate moiety of Shh binds within a hydrophobic pocket of Ptch1 (34, 35). Since unsaturation changes the geometry of the fatty acyl chain and the number of carbon atoms determines fatty acid chain length, we hypothesized that differential fatty acylation of Shh could impact Shh signaling output. To investigate this at the cellular level, we used Shh Light II cells, which stably express a Gli-1 responsive Firefly luciferase reporter along with constitutively expressed *Renilla* luciferase. Different fatty acylated forms of Shh were generated by incubating the recombinant, 19 kDa Shh protein with Hhat and a saturating concentration of individual NBD-labeled fatty acyl-CoAs (14:0, 16:0, 16:1 Δ9-10, 18:1 Δ9-10). Relative amounts of NBD-fatty acid incorporation were quantified, equal amounts of Shh protein were added to the cells, and EC_50_ curves were generated. Each of the four fatty acylated Shh species conferred more potent signaling compared to a mock reaction performed with unmodified Shh in the absence of NBD-acyl-CoA (Fig. 5*A*). The signaling potency of Shh modified with 14:0 and 16:0 fatty acids was ∼10 times higher than that of Shh modified with 16:1 and 18:1 fatty acids (Fig. 5*A* and Table 3). In the absence of Hhat, addition of NBD-acyl-CoAs to the reactions did not result in a shift of the dose–response curves beyond that of unacylated Shh (Fig. 5*B*). As expected, background levels of luciferase activity were obtained when Shh was omitted from the reaction (Fig. 5*C*). We conclude that Hhat-mediated differential fatty acylation regulates Shh signaling, with attachment of saturated fatty acids (myristate or palmitate) conferring higher signaling potency compared to monounsaturated fatty acids (palmitoleate or oleate).

**Table 3:** Signaling potency of NBD-fatty acylated Shh proteins

NBD-acyl-Shh	EC_50_ (μg/ml Shh)	Potency relative to NBD-16:0-Shh
Myristoyl (14:0)	0.04 ± 0.01	3.57×
Palmitoyl (16:0)	0.13 ± 0.02	1.00×
Palmitoleoyl (16:1)	0.89 ± 0.07	0.15×
Oleoyl (18:1)	1.80 ± 1.04	0.07×

Reported EC_50_ values represent the averages (±SD) of three titrations (n = 3) performed in duplicate (per fatty acylated Shh species).

## Discussion

### Development of a direct, fluorescent assay to measure Hhat activity

In this study, we used a fluorescence-based assay to perform extensive and previously unpursued kinetic analyses with purified Hhat. For many years, our laboratory relied on the use of radioactive palmitoyl-CoA analogs to assay Hhat, but safety considerations, cost effectiveness, and the nuisance of radioactive waste disposal prompted a switch to an alternative, less hazardous assay. NBD-palmitoyl-CoA has been used as a substrate in studies by our group and others for fatty acyltransferases including Hhat, DHHC17, DHHC20, and DGAT1 (25, 26, 27, 28, 29). The NBD fluorophore is located at the end of the acyl tail (Fig. 3*A*), farthest away from Hhat residues that have been shown to mediate catalysis at the CoA-acyl chain junction (12, 17). An N-terminal Shh peptide containing a C-terminal biotin served as the Shh recipient substrate and has also been used previously by our group and others in Hhat activity assays (6, 12, 14, 24). Incubation of these two substrates with purified Hhat resulted in a robust and reproducible assay. Moreover, kinetic data obtained with NBD-palmitoyl-CoA and the Shh peptide (Fig. 1, *E* and *F*) were in good agreement with those obtained with ^3^H-palmitoyl-CoA and a similar Shh peptide (12). Importantly, the presence of an NBD group did not shield apparent kinetic differences for Hhat among fatty acyl-CoAs with differing acyl chains (Fig. 4 and Table 2).

### Kinetic mechanism of Hhat catalysis

To date, the kinetic mechanism of Hhat has not been determined. Hhat binds Shh in the luminal leaflet of the ER membrane and palmitoyl-CoA in the cytoplasmic leaflet (12, 17), suggesting that binding of the two substrates might be independent. Here, we show that Hhat palmitoylates Shh *via* a random sequential mechanism, in which Shh and palmitoyl-CoA bind in no obligate order to form a ternary complex with Hhat. This is consistent with evidence in the literature that Hhat can bind palmitoyl-CoA in the absence of Shh (25) and that Hhat can bind Shh in the absence of palmitoyl-CoA (17). In binding assays, the latter study also found that binding of Shh to Hhat was three times higher in the presence of palmitoyl-CoA, indicating that palmitoyl-CoA binding enhances Shh binding. However, under our assay conditions, the observed patterns in Lineweaver–Burk plots (intersection point of lines was not above the X-axis, Fig. 2, *A* and *B*) were indicative of lack of positive cooperativity (31), suggesting that neither substrate enhanced the binding of the other.

An important, unresolved question regarding Shh acylation is whether Hhat directly N-acylates the N-terminal cysteine or whether catalysis proceeds *via* initial S-acylation of the cysteine sulfhydryl, followed by an intramolecular S-to-N shift. Our data indicate that a Shh peptide with a blocked, N-acetylated N terminus does not serve as an Hhat substrate (Fig. 1*D*), despite the presence of a free sulfhydryl group on the N-terminal cysteine. This confirms results we obtained using a radioactivity-based Hhat assay (6) and supports a direct, one-step mechanism for N-acylation, rather than a two-step, S-to-N shift. Direct amide linkage is also favored based on Hhat structural analysis, whereby D339 is proposed to serve as a base to activate the N-terminal amine for nucleophilic attack of the carbonyl carbon of the palmitoyl-CoA molecule (12).

### Fatty acyl-CoA substrate specificity of Hhat

Several lines of evidence indicate that the fatty acyl-CoA substrate specificity of Hhat is not limited to palmitoyl-CoA. Addition of unlabeled fatty acyl-CoAs ranging in chain length from 10 to 18 carbons inhibited incorporation of ^125^I-Iodopalmitate into Shh by purified Hhat (6), implying that Hhat might be able to bind to a variety of fatty acyl-CoAs. Furthermore, when Shh was purified from mammalian cells, N-terminal acyl attachments to Shh other than palmitate (16:0) were detected by mass spectrometry, including 14:0, 14:1, 16:1, 18:0, 18:1, and 20:4 fatty acids (19, 20). The prevalence of various fatty acyl attachments on Shh was found to be dependent upon the metabolic state and Shh expression level within cells (19). Mammalian cells and tissues contain multiple fatty acids and acyl-CoAs, with 16:0, 18:0, 18:1, and 18:2 being the most abundant (36, 37, 38, 39, 40). Thus, Hhat would have access to multiple fatty acyl-CoAs in potentially equal abundance *in vivo*. Hhat is the only known acyltransferase for Shh, and autoacylation of Shh in cells is highly unlikely given that Shh and acyl-CoAs are compartmentalized on opposite sides of the ER membrane and that the concentrations of free acyl-CoAs are very low (41). Thus, we hypothesized that Hhat is able to utilize multiple acyl-CoA substrates, a supposition confirmed by the experiments depicted in Figures 3 and 4.

Other fatty acyltransferases have been shown to utilize more than one fatty acyl-CoA as a substrate. For example, members of the DHHC acyltransferase family have substrate binding pockets that function as “molecular rulers,” allowing binding and utilization of different chain length fatty acids (18, 37, 42). Additionally, while oleoyl-CoA serves as the primary acyl-CoA substrate for DGAT1, with the highest apparent turnover rate, *in vitro* kinetic analyses with purified hDGAT1 revealed that DGAT1 demonstrates greatest apparent affinity for 16-carbon fatty acyl-CoAs among several fatty acyl-CoAs tested (43). Moreover, saturated and monounsaturated fatty acids ranging from 10 to 16 carbons, with a *cis-* but not *trans-*double bond at Δ9, can serve as substrates for Porcn-mediated fatty acylation of Wnt proteins in cells (44, 45). Differences in cryo-EM structures observed between the acyl-CoA binding cavity of Hhat and other MBOATs led to the proposition that Hhat’s acyl-CoA binding pocket is more confined, with a preference for palmitoyl-CoA (12, 17) and restriction of the pocket to a maximum of 16 carbons (17). However, our biochemical analyses of purified Hhat reveal not only a substrate range wider than palmitoyl-CoA but also that the enzyme can accommodate an iodine (6, 24) or an NBD group (this study) on the end of the fatty acyl chain and transfer the labeled acyl chain to Shh. It is possible that the use of antibodies (Fab fragments in (12) or megabodies in (17)) to generate the Hhat structures conferred additional rigidity to the enzyme that is not otherwise present when the enzyme is not subjected to cryo-EM conditions. Alternatively, the use of different detergents may allow Hhat to adopt different conformations, enabling flexibility to bind different fatty acyl-CoAs. Other studies have demonstrated that MBOATs, including Hhat, can be differentially active (14) and adopt different oligomeric states (43) in different detergents.

Our data indicate that palmitoleoyl-CoA and oleoyl-CoA are substrates for Hhat. This is consistent with the findings that these unsaturated fatty acids are attached to Shh in cells and are even the major species of acylated Shh under certain cellular conditions (19, 45). The kink in palmitoleoyl-CoA conferred by monounsaturation made no apparent impact on the kinetic profiles when compared to palmitoyl-CoA (Fig. 4). A previous study by our group found that when assayed in microsomal membranes, Hhat did not utilize [^125^I]iodo-pentadecenoyl-CoA, a CoA with a 15-carbon cis-9 monounsaturated, iodinated fatty acid, as a substrate (46). It is possible that the use of different detergents and enzyme environments may account for the result in the latter study. Overall, the data presented in this study indicate that Hhat can attach a spectrum of fatty acids to Shh. Given this finding and that differential fatty acylation of Shh has been reported to be dependent upon cell context (19), it is possible that Shh signaling in cells which have altered metabolic states and/or high levels of Shh expression (*e.g.*, cancer cells) might be mediated by Shh proteins modified with fatty acids other than palmitate.

### Consequences of differential fatty acylation Shh on cellular signaling

Our findings, and those of others, indicate that heterogeneous fatty acylation impacts Shh signaling (21, 22). Our results are consistent with a previous report that hydrophobic modifications to Shh confer greater signaling potency compared to unmodified Shh and that signaling conferred by myristoylated Shh is ∼four times more potent compared to palmitoylated Shh (21). Here, we show that the potency of signaling output was stronger when Shh was modified with saturated compared to monounsaturated fatty acids. Only one other study examined the effect of monounsaturation on cellular signaling, where it was shown that Shh isolated from chick limb buds exhibits reduced signaling activity when the explants are incubated with palmitoleate compared to palmitate (19). We have shown in a direct Gli-1 reporter system with two unsaturated fatty acyl-CoAs that the degree of saturation of the fatty acid chain regulates Shh signaling.

Multiple stages of the Shh signaling pathway in cells are likely to be impacted by differential fatty acylation. Dually lipidated Shh associates with the cell membrane and is then secreted from the cell in a process mediated by SCUBE2 and Dispatched (47). Shh travels to Ptch on receiving cells *via* carrier proteins (48). Any and all of these steps in the signaling pathway may be regulated by the particular acyl attachment on Shh. Indeed, differential fatty acylation of Shh has been shown to alter lipid raft association and secretion of Shh (19). Furthermore, while interaction of SCUBE2 with the palmitate moiety is not absolutely required for Shh binding and secretion from cells, the presence of palmitate enhances these activities (49). After secretion, GAS1 acts as the final agent (in a sequence with other preceding hand-off proteins CDON/BOC) to deliver dually lipidated Shh to Ptch1 on the receiving cell surface (48, 50). Although not absolutely required for binding of Shh to Ptch1 and subsequent signaling, GAS1-mediated handoff of Shh greatly enhances these activities, and interaction between GAS1 and Shh requires a Shh N-terminal fatty acid (48, 50). Importantly, the affinity of GAS1 for Shh is ∼10-fold higher when palmitate is the N-terminal modification compared to octanoate (8:0) (50). The final Shh binding partner in the pathway that mediates signaling is Ptch, whose inhibition and subsequent activation of Smo are robustly induced by binding of palmitoylated Shh (20, 33). Recent structural studies of Ptch1 have revealed that Ptch1 is a dimer (Ptch1-A,B); the N-terminal palmitate of Shh binds within the Ptch1-A monomer, whereas the globular portion of Shh binds within the Ptch1-B monomer (34, 35). This structural revelation explains the previously puzzling findings that Shh lacking a fatty acyl attachment exhibited compromised signaling yet was able to bind Ptch1 (20, 33). Interaction between dually lipidated Shh and a putative cholesterol transport tunnel in Ptch1A has been proposed to either stabilize Ptch1 or block cholesterol transport (51), thereby inactivating Ptch. When Ptch1 is bound by palmitoylated Shh, cholesterol in the plasma membrane becomes accessible to activate Smo (52). Whether and how differential acylation might specifically impact Shh binding to Ptch has only been explored in one study, using cells overexpressing a truncated Ptch protein and two different hydrophobically modified forms of Shh (21). No difference in binding affinity of the two modified Shh species to Ptch was detected under these conditions, but the authors posited that high levels of receptor overexpression may have masked potential differences. Thus, additional *in vitro* experiments to directly test the affinity of differentially acylated Shh for its various binding partners would be extremely informative in further characterizing the role of fatty acylation in Shh signaling.

## Experimental procedures

### Reagents

n-Dodecyl-β-D-maltopyranoside was purchased from Anatrace (catalog # D310). n-Octyl-β-D-glucopyranoside (OG) was purchased from EMD Millipore (catalog # 494459). Triton X-100 was purchased from Fisher Scientific (catalog # BP151–500). MES was purchased from Fisher Scientific (catalog # BP300–100). DTT was purchased from Promega (catalog # V3151). Anti-FLAG M2 Affinity Gel (catalog # A2220), and 3× FLAG peptide (catalog # F4799) were purchased from Millipore Sigma. Pierce High Capacity Streptavidin Beads were purchased from Thermo Scientific (catalog # 20361). Antibodies were purchased as follows: rabbit α-HA (Sigma, catalog #H6908), mouse α-Shh E-1 (Santa Cruz, catalog # sc-365112), ECL donkey anti-rabbit IgG, horseradish peroxidase linked whole antibody (Cytiva, catalog # NA934), and ECL sheep anti-mouse IgG, horseradish peroxidase linked whole antibody (Cytiva, catalog # NA931). NBD-palmitoyl-CoA, NBD-oleoyl-CoA, and NBD-palmitic acid were obtained from Avanti Polar Lipids. NBD-myristoyl-CoA and NBD-palmitoleoyl-CoA were synthesized by the Memorial Sloan Kettering Organic Synthesis Core Lab. Biotinylated Shh peptides (CGPGRGFGKR-(PEG2)-K(Biotin)-NH_2_) and (AGPGRGFGKR-(PEG2)-K(Biotin)-NH_2_) were synthesized by Anaspec and Peptide 2.0.

### Cell culture

HEK293FT cells (passage 3–5) were cultured at 37 °C in Dulbecco's Modified Eagle Medium (4500 g/L glucose, 1% penicillin, 1% streptomycin) containing 1× Glutamax, 1 mM sodium pyruvate, 1× MEM Non-Essential Amino Acids Solution, 10% fetal bovine serum, 0.5 g/L geneticin (G418). Shh Light II cells were cultured at 37 °C in Dulbecco's Modified Eagle Medium (as above) with 10% calf serum, 1× Glutamax, 0.4 g/L G418, and 0.15 g/L zeocin. Glutamax (catalog # 35050061), sodium pyruvate (catalog # 11360070), and 1× MEM Non-Essential Amino Acids Solution (catalog # 11140050) were purchased from Gibco.

### Hhat Purification

Hhat was purified as reported previously (6, 23), with a few modifications. 100 mm plates of 85% confluent HEK293FT cells were transfected with triple tagged (HA-FLAG-His) WT or pcDNA3.1 empty vector (6 μg DNA, 15 μl Lipofectamine 2000 Transfection Reagent [Invitrogen catalog # 11668019], and 1 ml Opti-MEM media [Gibco catalog # 31985070] per plate). Eighteen to 24 h posttransfection, cells were split 1:2, then incubated for an additional 24 h. Cells were collected in ice-cold STE buffer by gentle scraping, pelleted by centrifugation at 1000*g* for 10 min, and then washed with ice-cold STE buffer. Cell pellets were resuspended in ice-cold hypotonic lysis buffer (10 mM Hepes, pH 7.3 and 0.2 mM MgCl_2_) and incubated on ice for 15 min. Cells were lysed by 30 up/down strokes in a Dounce homogenizer on ice, sucrose was added to a final concentration of 0.25 mM, and membranes were pelleted by ultracentrifugation at 100,000*g* for 45 min (4 °C) in a Beckman Ti-70.1 fixed angle rotor. After centrifugation, the supernatant was discarded, and the membrane pellets were resuspended by Dounce homogenization in hypotonic lysis buffer supplemented with 0.25 mM sucrose on ice and pelleted by ultracentrifugation as above. Membrane pellets were resuspended in ice-cold solubilization buffer (20 mM Hepes, pH 7.5, 350 mM NaCl, 1% [v/v] glycerol, and 1% [w/v] n-dodecyl-β-D-maltopyranoside) in a Dounce homogenizer on ice and solubilized by rocking for 1 h at 4 °C. Ultracentrifugation as above was repeated to pellet the insoluble material, which was subsequently discarded. Anti-FLAG M2 Affinity Gel, which had been washed twice and resuspended in solubilization buffer, was added to the detergent-solubilized membranes, and the mixture was rocked overnight at 4 °C. The next morning, the FLAG M2 beads were pelleted by centrifugation at 1000*g* for 2 min and washed four times with solubilization buffer. Elution buffer (solubilization buffer supplemented with 150 μg/ml 3× FLAG peptide) was added, and tubes were rocked at 4 °C for 2 h. Beads were pelleted by centrifugation at 1000*g* for 2 min, and eluate was collected from each tube. This elution process was repeated twice with decreasing volumes of elution buffer, and all eluates were combined. Protein concentrations were calculated using an extinction coefficient for Hhat of 187,085 cm^-1^M^-1^ and absorbance read at 280 nm (empty vector eluate was used as the blank).

### In Vitro Hhat assay

NBD-acyl-CoA, biotinylated Shh peptide, purified Hhat (typically 130 nM), and reaction buffer (final concentrations of 142 mM MES, pH 6.5, 0.07% [w/v] OG, and 850 μM DTT) in a volume of 100 μl were added at the indicated concentrations to 0.5 ml tubes at room temperature. Reactions were initiated by addition of Hhat, and tubes were incubated at 37 °C in the dark for the indicated amount of time. Reactions were quenched by placement of tubes on ice, followed by addition of a 60 μl cold suspension of Pierce High Capacity Streptavidin Beads in wash buffer (167 mM MES, pH 6.5 and 0.083% [v/v] Triton X-100). Tubes were rocked at 4 °C for 1 h. Beads were centrifuged at 2700*g*, 4 °C in a microcentrifuge for 1 min, then washed three times with 100 μl wash buffer. After the final wash, beads were transferred in 60 μl wash buffer to wells of a black side, clear bottom 96-well plate (Thermo Scientific catalog # 265301), and fluorescence was read in a BioTek Synergy H1 plate reader at emission/excitation wavelengths of 465/535 nm using Gen5 2.09 software. All assays were performed at least twice in duplicate. For determination of the stoichiometry of palmitoylation, reactions contained 10 μM Shh peptide and 10 μM NBD-palmitoyl-CoA and were incubated at 37 °C for 90 min. This experiment was performed twice in duplicate, and the reported error is SD.

### NBD-fatty acylation of Shh protein

Recombinant, 19 kDa Shh protein (Cys24-Gly197), purified from *E. coli* as described previously (6, 23), was incubated for 1 h with reaction buffer, purified Hhat, and individual NBD-acyl-CoAs in the reaction conditions described above. Immediately following the reaction, sample buffer was added, samples were boiled at 95 °C for 5 min, and 32% (volume) of each sample was subjected to electrophoresis in 15% polyacrylamide gels. In-gel NBD fluorescence was detected on a Typhoon FLA 9500 Imager using a 473 nm laser and excitation/emission settings of 495/519 nm. For subsequent Western blotting analyses, proteins were transferred from the gel to PVDF, the membrane was blocked with 5% [w/v] nonfat, dry milk in TBST (20 mM Tris, 150 mM NaCl, 0.1% [v/v] Tween-20) for 10 min, then incubated overnight at 4 °C with primary antibodies (for Hhat, 1:1000 rabbit α-HA; for Shh, 1:200 mouse α-Shh E-1) in TBST, 5% [w/v] nonfat, dry milk. The next day, blots were washed three times for 5 min with TBST, then incubated at room temperature for 1.5 h with a 1:2500 dilution of the appropriate (anti-rabbit or anti-mouse) horseradish peroxidase–conjugated secondary antibody in TBST, 5% [w/v] nonfat, dry milk. Blots were washed three times for 5 min with TBST, developed with Pierce ECL Western Blotting Substrate (ThermoFisher catalog #32209), and imaged on a ChemiDoc Gel Imaging System (Bio-Rad). Relative band intensities were quantified using ImageJ software. NBD-fatty acylated Shh band intensities were normalized to Shh band intensities on the Western blot. This experiment was performed four times.

### Hhat kinetic analysis

Kinetic analysis was carried out by *in vitro* acylation assays performed as described above, with a 10-min reaction incubation period. Background RFU values from matched reactions containing elution buffer instead of Hhat were subtracted for each assay condition. The initial rate of Hhat-mediated NBD-acylation of Shh was quantified as pmol NBD-acylated Shh per minute per pmol Hhat and reported in (min^-1^). Assays were performed at least twice in duplicate. Titration data were graphed in GraphPad Prism 9 software. Data from substrate titrations performed in the presence of various fixed concentrations of cosubstrate were fit to the Michaelis–Menten model (V = V_max_[S]/(K_m_ + [S])), from which apparent K_m_ and k_cat_ values were derived. To obtain the apparent K_i_ for RU-SKI 201, data from titration of NBD-palmitoyl-CoA in the presence of various fixed concentrations of RU-SKI 201 were fit to the competitive inhibitor equation (V = V_max_[S]/([S] + K_m_(1+[I]/K_i_)). Lineweaver–Burk plots (1/V = K_m_/V_max_[S] + 1/V_max_) were generated *via* transformation of titration data to double-reciprocal values and simple linear regression.

### Shh signaling assays

Approximately 100,000 Shh Light II cells per well were seeded in 96-well tissue culture plates with a white side and clear bottom (Corning Costar catalog # 3610) and cultured until confluent. 40 μM recombinant, 19 kDa Shh protein (Cys24-Gly197) purified from *E. coli* (6, 23) was incubated with reaction buffer (final concentrations of 108 mM MES, pH 6.5, 0.05% [w/v] OG, 645 μM DTT), 200 μM NBD-fatty acyl-CoA, and 170 nM purified Hhat (or equal volume of elution buffer in Hhat-lacking reactions), in a total volume of 100 μl at 37 °C for 2 h. Reactions were quenched on ice. To quantify the relative amount of NBD-fatty acylated Shh in each sample, 30% (volume) of each reaction was mixed with sample buffer, incubated at 95 °C for 5 min, then subjected to electrophoresis in 12% polyacrylamide gels (Bio-Rad catalog # 4561044). Relative in-gel NBD fluorescence was detected and quantified as above, using average values from two gels/quantifications. Dilutions containing equal amounts of Shh protein from each reaction were prepared in low-serum media (Dulbecco's Modified Eagle Medium, 4500 g/L glucose, 1% penicillin, 1% streptomycin, with 0.5% calf serum, 1× Glutamax, 0.4 g/L G418, and 0.15 g/L zeocin). For control reactions lacking Shh protein, dilutions were prepared using the equivalent dilution factor to Shh-containing reactions. Media in the wells were replaced with 100 μl of prepared reaction dilutions, and plates were incubated at 37 °C for ∼42 h. Cells were then lysed, and Firefly and *Renilla* luciferase activities were measured using the Promega Dual-Luciferase Reporter Assay System (Promega catalog # E1980), according to the manufacturer’s instructions. Titrations were performed two or three times (as indicated in the figure legend) in duplicate (two wells of cells per reaction dilution). The Firefly/*Renilla* luciferase ratio for each well was calculated, and data were normalized as indicated in the figure legend. Titration data were graphed using GraphPad Prism 9 software and fit to the curve [Agonist] *versus* response (three parameters) (Y = Bottom + X(Top - Bottom)/(EC_50_ + X)), from which EC_50_ values were derived. This entire experiment was repeated and resulted in the same outcome.

## Data availability

All data are contained within the manuscript.

## Conflict of interest

The authors declare they have no conflicts of interest with the contents of this article.

## References

[1] Kong J.H., Siebold C., Rohatgi R. (2019). Biochemical mechanisms of vertebrate hedgehog signaling. Development.

[2] Petrova R., Joyner A.L. (2014). Roles for Hedgehog signaling in adult organ homeostasis and repair. Development.

[3] Qi X., Li X. (2020). Mechanistic insights into the generation and transduction of hedgehog signaling. Trends Biochem. Sci..

[4] Mann R.K., Beachy P.A. (2004). Novel lipid modifications of secreted protein signals. Annu. Rev. Biochem..

[5] Chamoun Z., Mann R.K., Nellen D., von Kessler D.P., Bellotto M., Beachy P.A. (2001). Skinny Hedgehog, an acyltransferase required for palmitoylation and activity of the hedgehog signal. Science.

[6] Buglino J.A., Resh M.D. (2008). Hhat is a palmitoylacyltransferase with specificity for N-palmitoylation of Sonic Hedgehog. J. Biol. Chem..

[7] Resh M.D. (2021). Palmitoylation of hedgehog proteins by hedgehog acyltransferase: roles in signalling and disease. Open Biol..

[8] Micchelli C.A., The I., Selva E., Mogila V., Perrimon N. (2002). Rasp, a putative transmembrane acyltransferase, is required for Hedgehog signaling. Development.

[9] Chen M.H., Li Y.J., Kawakami T., Xu S.M., Chuang P.T. (2004). Palmitoylation is required for the production of a soluble multimeric Hedgehog protein complex and long-range signaling in vertebrates. Genes Dev..

[10] Jiang J., Hui C.C. (2008). Hedgehog signaling in development and cancer. Dev. Cell.

[11] Wu F., Zhang Y., Sun B., McMahon A.P., Wang Y. (2017). Hedgehog signaling: from basic biology to cancer therapy. Cell Chem. Biol..

[12] Jiang Y., Benz T.L., Long S.B. (2021). Substrate and product complexes reveal mechanisms of Hedgehog acylation by HHAT. Science.

[13] Lanyon-Hogg T., Patel N.V., Ritzefeld M., Boxall K.J., Burke R., Blagg J. (2017). Microfluidic mobility shift assay for real-time analysis of peptide N-palmitoylation. SLAS Discov..

[14] Lanyon-Hogg T., Masumoto N., Bodakh G., Konitsiotis A.D., Thinon E., Rodgers U.R. (2015). Click chemistry armed enzyme-linked immunosorbent assay to measure palmitoylation by hedgehog acyltransferase. Anal. Biochem..

[15] Lanyon-Hogg T., Ritzefeld M., Sefer L., Bickel J.K., Rudolf A.F., Panyain N. (2019). Acylation-coupled lipophilic induction of polarisation (Acyl-cLIP): a universal assay for lipid transferase and hydrolase enzymes. Chem. Sci..

[16] Andrei S.A., Tate E.W., Lanyon-Hogg T. (2022). Evaluating hedgehog acyltransferase activity and inhibition using the acylation-coupled lipophilic induction of polarization (Acyl-cLIP) assay. Met. Mol. Biol..

[17] Coupland C.E., Andrei S.A., Ansell T.B., Carrique L., Kumar P., Sefer L. (2021). Structure, mechanism, and inhibition of Hedgehog acyltransferase. Mol. Cell.

[18] Jennings B.C., Linder M.E. (2012). DHHC protein S-acyltransferases use similar ping-pong kinetic mechanisms but display different acyl-CoA specificities. J. Biol. Chem..

[19] Long J., Tokhunts R., Old W.M., Houel S., Rodgriguez-Blanco J., Singh S. (2015). Identification of a family of fatty-acid-speciated sonic Hedgehog proteins, whose members display differential biological properties. Cell Rep..

[20] Pepinsky R.B., Zeng C., Wen D., Rayhorn P., Baker D.P., Williams K.P. (1998). Identification of a palmitic acid-modified form of human Sonic Hedgehog. J. Biol. Chem..

[21] Taylor F.R., Wen D., Garber E.A., Carmillo A.N., Baker D.P., Arduini R.M. (2001). Enhanced potency of human Sonic hedgehog by hydrophobic modification. Biochemistry.

[22] Baker D.P., Taylor F.R., Pepinsky R.B. (2007). Purifying the hedgehog protein and its variants. Met. Mol. Biol..

[23] Asciolla J.J., Rajanala K., Resh M.D. (2019). *In vitro* analysis of hedgehog acyltransferase and porcupine fatty acyltransferase activities. Met. Mol. Biol..

[24] Petrova E., Rios-Esteves J., Ouerfelli O., Glickman J.F., Resh M.D. (2013). Inhibitors of Hedgehog acyltransferase block sonic hedgehog signaling. Nat. Chem. Biol..

[25] Asciolla J.J., Resh M.D. (2019). Hedgehog acyltransferase promotes uptake of palmitoyl-CoA across the endoplasmic reticulum membrane. Cell Rep..

[26] Rojas A., Del Campo J.A., Clement S., Lemasson M., Garcia-Valdecasas M., Gil-Gomez A. (2016). Effect of quercetin on hepatitis C virus life cycle: from viral to Host targets. Sci. Rep..

[27] McFie P.J., Banman S.L., Kary S., Stone S.J. (2011). Murine diacylglycerol acyltransferase-2 (DGAT2) can catalyze triacylglycerol synthesis and promote lipid droplet formation independent of its localization to the endoplasmic reticulum. J. Biol. Chem..

[28] Rana M.S., Kumar P., Lee C.J., Verardi R., Rajashankar K.R., Banerjee A. (2018). Fatty acyl recognition and transfer by an integral membrane S-acyltransferase. Science.

[29] Verardi R., Kim J.S., Ghirlando R., Banerjee A. (2017). Structural basis for substrate recognition by the ankyrin repeat domain of human DHHC17 palmitoyltransferase. Structure.

[30] Resh M.D., Glickman J.F., Petrova E., Ouerfelli O. (2016).

[31] Segel I.H. (1975).

[32] Lanyon-Hogg T., Ritzefeld M., Zhang L., Andrei S.A., Pogranyi B., Mondal M. (2021). Photochemical probe identification of a small-molecule inhibitor binding site in hedgehog acyltransferase (HHAT). Angew. Chem. Int. Ed. Engl..

[33] Tukachinsky H., Petrov K., Watanabe M., Salic A. (2016). Mechanism of inhibition of the tumor suppressor patched by Sonic Hedgehog. Proc. Natl. Acad. Sci. U. S. A..

[34] Qian H., Cao P., Hu M., Gao S., Yan N., Gong X. (2019). Inhibition of tetrameric patched1 by sonic hedgehog through an asymmetric paradigm. Nat. Commun..

[35] Rudolf A.F., Kinnebrew M., Kowatsch C., Ansell T.B., El Omari K., Bishop B. (2019). The morphogen Sonic Hedgehog inhibits its receptor patched by a pincer grasp mechanism. Nat. Chem. Biol..

[36] Spector A.A., Yorek M.A. (1985). Membrane lipid composition and cellular function. J. Lipid Res..

[37] Greaves J., Munro K.R., Davidson S.C., Riviere M., Wojno J., Smith T.K. (2017). Molecular basis of fatty acid selectivity in the zDHHC family of S-acyltransferases revealed by click chemistry. Proc. Natl. Acad. Sci. U. S. A..

[38] Woldegiorgis G., Spennetta T., Corkey B.E., Williamson J.R., Shrago E. (1985). Extraction of tissue long-chain acyl-CoA esters and measurement by reverse-phase high-performance liquid chromatography. Anal. Biochem..

[39] Hama K., Fujiwara Y., Takashima S., Hayashi Y., Yamashita A., Shimozawa N. (2020). Hexacosenoyl-CoA is the most abundant very long-chain acyl-CoA in ATP binding cassette transporter D1-deficient cells. J. Lipid Res..

[40] Palladino A.A., Chen J., Kallish S., Stanley C.A., Bennett M.J. (2012). Measurement of tissue acyl-CoAs using flow-injection tandem mass spectrometry: Acyl-CoA profiles in short-chain fatty acid oxidation defects. Mol. Genet. Metab..

[41] Faergeman N.J., Knudsen J. (1997). Role of long-chain fatty acyl-CoA esters in the regulation of metabolism and in cell signalling. Biochem. J..

[42] Rana M.S., Lee C.J., Banerjee A. (2019). The molecular mechanism of DHHC protein acyltransferases. Biochem. Soc. Trans..

[43] Wang L., Qian H., Nian Y., Han Y., Ren Z., Zhang H. (2020). Structure and mechanism of human diacylglycerol O-acyltransferase 1. Nature.

[44] Rios-Esteves J., Resh M.D. (2013). Stearoyl CoA desaturase is required to produce active, lipid-modified Wnt proteins. Cell Rep..

[45] Tuladhar R., Yarravarapu N., Ma Y., Zhang C., Herbert J., Kim J. (2019). Stereoselective fatty acylation is essential for the release of lipidated WNT proteins from the acyltransferase Porcupine (PORCN). J. Biol. Chem..

[46] Asciolla J.J., Miele M.M., Hendrickson R.C., Resh M.D. (2017). An *in vitro* fatty acylation assay reveals a mechanism for Wnt recognition by the acyltransferase Porcupine. J. Biol. Chem..

[47] Tukachinsky H., Kuzmickas R.P., Jao C.Y., Liu J., Salic A. (2012). Dispatched and scube mediate the efficient secretion of the cholesterol-modified hedgehog ligand. Cell Rep..

[48] Wierbowski B.M., Petrov K., Aravena L., Gu G., Xu Y., Salic A. (2020). Hedgehog pathway activation requires coreceptor-catalyzed, lipid-dependent relay of the sonic hedgehog ligand. Dev. Cell.

[49] Creanga A., Glenn T.D., Mann R.K., Saunders A.M., Talbot W.S., Beachy P.A. (2012). Scube/You activity mediates release of dually lipid-modified Hedgehog signal in soluble form. Genes Dev..

[50] Huang P., Wierbowski B.M., Lian T., Chan C., Garcia-Linares S., Jiang J. (2022). Structural basis for catalyzed assembly of the Sonic Hedgehog-Patched1 signaling complex. Dev. Cell.

[51] Kinnebrew M., Luchetti G., Sircar R., Frigui S., Viti L.V., Naito T. (2021). Patched 1 reduces the accessibility of cholesterol in the outer leaflet of membranes. Elife.

[52] Kowatsch C., Woolley R.E., Kinnebrew M., Rohatgi R., Siebold C. (2019). Structures of vertebrate patched and smoothened reveal intimate links between cholesterol and Hedgehog signalling. Curr. Opin. Struct. Biol..

[53] Smith R.H., Powell G.L. (1986). The critical micelle concentration of some physiologically important fatty acyl-coenzyme A's as a function of chain length. Arch. Biochem. Biophys..

[54] Constantinides P.P., Steim J.M. (1985). Physical properties of fatty acyl-CoA. Critical micelle concentrations and micellar size and shape. J. Biol. Chem..

